# “I Will Experience This Trauma Over and Over Again”: Sexual and Gender-Based Violence, Forced Migration and Structural Violence

**DOI:** 10.1177/08862605251338785

**Published:** 2025-06-03

**Authors:** Jenny Phillimore, Karen Block, Hannah Bradby, Hoayda Darkal, Lisa Goodson, Anna Papoutsi, Cathy Vaughan

**Affiliations:** 1 University of Birmingham, UK; 2 University of Melbourne, VIC, Australia; 3 University of Uppsala, Sweden; 4 University of Plymouth, UK

**Keywords:** forced migration, structural violence, women, sexual and gender-based violence, power, interpersonal violence

## Abstract

Forced migration has reached unprecedented levels as millions are forced to seek refuge from conflict, persecution, and violence. This exodus includes women enduring the traumas of displacement alongside sexual and gender-based violence (SGBV). Upon reaching supposed places of refuge, they encounter the structural violence of immigration and asylum regimes. Against this backdrop, the intersection of SGBV, forced migration, and structural violence emerges as an urgent area of study. Drawing from extensive qualitative interviews in Australia, Sweden, and the United Kingdom, we set out to examine the impact of structural violence on the lives of forced migrant survivors of SGBV. The article introduces a novel framework to analyze how SGBV, forced migration and structural violence intersect and impact on the lives of survivors. The framework synthesizes (a) the intimate violence of dependency, (b) the slow violence of everyday life, and (c) the gender insensitivity characteristic of determination regimes. Survivors endure a range of injustices: the intimate violence of dependency traps women in controlling relationships; the asylum system’s slow violence leaves them in substandard and undignified conditions; and gender-insensitivity renders their SGBV experiences invisible, often retraumatizing survivors. Within this framework, we describe how these intersecting forms of structural violence underpinning immigration systems, systematically fail those at risk of SGBV, rendering them vulnerable to interpersonal violence instead of protecting them. We call for immigration and asylum systems to prioritize the protection and well-being of women, many of whom are SGBV survivors. As forced migrants face increasingly hostile statutory regimes, we must recognize and address the structural violence that perpetuates harm and denies them protection. Failure to act risks further perpetuating the cycle of violence, trauma and injustice, undermining principles of safety and refuge for those in dire need.

## Introduction

The number of forcibly displaced people has reached an all-time high (UNHCR, 2021a). While most are internally displaced or reside in adjacent countries, millions arrive in high-income countries as refugees or seeking asylum (UNHCR, 2021a). They often experience violence across a continuum transcending time and place from pre-conflict, through displacement, and then on lengthy journeys across multiple borders (Hourani et al., 2021). While UNHCR envisages resettlement as offering durable protection (Jubilut and de Lima Madureira, 2016, np), forced migrants regularly face structural violence through hostile immigration and asylum systems that exposure them to racism and exclusion (Canning, 2017; Hourani et al., 2021). Higher-income countries have introduced increasingly harsh asylum regimes, at the same time fueling and responding to increasing public hostility toward asylum seekers flaunted as “illegal immigrants,” while restrictive visa systems and deadly geographies deter arrivals. Asylum processes that are unjust and cruel (Canning, 2017) compound the trauma for women who have experienced sexual and gender-based violence (SGBV) before and during mobility (Hourani et al., 2021) and render them vulnerable to further abuse (Keygnaert et al., 2012).

The article adopts a structural violence approach (Henderson, 2022) toward the experiences of women forced migrant survivors of SGBV in Australia, Sweden, and the United Kingdom, to explore how the structural violence of immigration and asylum regimes impact on their lives. We introduce a new analytical framework combining three forms of structural violence: the intimate violence of dependency; slow violence of the everyday (Mayblin et al., 2020); and gender-insensitivity of asylum determination regimes. Due to these interacting forms of structural violence (Galtung, 1969) or “multi-axial models of suffering,” (Farmer, 1996, p. 272), women, who are widely acknowledged to be extremely vulnerable (UNHCR, 2003) and at risk of interpersonal violence, are routinely failed. Based on interviews with survivors, we examine accounts of the structural violence they faced once under state protection. We show how this structural violence resulted in further harms including SGBV victimization. This article consists of introductions to how SGBV and structural violence are intertwined with forced migration, followed by a description of how (a) the violence of dependency, (b) slow violence, and (c) gender insensitivity contribute to the harms of interpersonal violence. We then illustrate how these three forms of structural violence play out in the lives of forced migrant women subject to SGBV in the United Kingdom, Sweden, and Australia.

### SGBV and Forced Migration

Forced displacement has doubled in the past decade and is predicted to further intensify (UNHCR, 2021a). The term forced migrant refers to all those subject to coerced migratory movement (IOM, 2011) and includes asylum seekers, rejected asylum seekers, undocumented people, refugees, and those forced to marry or join forced migrants on spousal (Taha, 2021). Refugee reception in countries of refuge largely depends on entry route: those resettled directly through specific schemes are deemed “good” refugees, while spontaneous sanctuary seekers are frequently vilified and denied access to many services (Mountz, 2016). Thus, how migrants cross borders shapes their treatment in refuge, although they all may face racism and structural inequalities (Hourani et al., 2021). UNHCR and IDC (2016) argues that vulnerability criteria should be applied in determining protection and assistance for forced migrants, recognizing that women, survivors of violence and torture, and those with health conditions, require specific support. SGBV is a frequent but hidden aspect of refugee displacement, with women survivors embodying multiple intersecting forms of vulnerability (Freedman, 2016), deserving special protection (UNHCR & IDC, 2016).

Some 48% of forced migrants are women (UNHCR, 2021a). While the proportion experiencing SGBV is unknown because of poor reporting mechanisms and fears surrounding disclosure (Papoutsi et al., 2022), it is thought to exceed 60% (Keygnaert et al., 2012). Gender persecution can be a catalyst for flight (UNHCR, 2021b), with SGBV commonplace during conflict (Spangaro et al., 2015). Some forms of SGBV, including intimate partner violence (IPV) and forced marriage, increase in displacement (Marsh et al., 2006). It is well documented that SGBV occurs across the refugee journey, yet each stage tends to be studied in isolation (Hourani et al., 2021). Instead, as argued by some scholars, a continuum of violence occurring across multiple places over time (Freedman, 2016) is a more appropriate framework. Little work has acknowledged that SGBV can continue into refuge, and that in refuge, women can face interpersonal violence and structural violence by the states responsible for their protection.

### Structural Violence and Forced Migration

**Figure 1.**
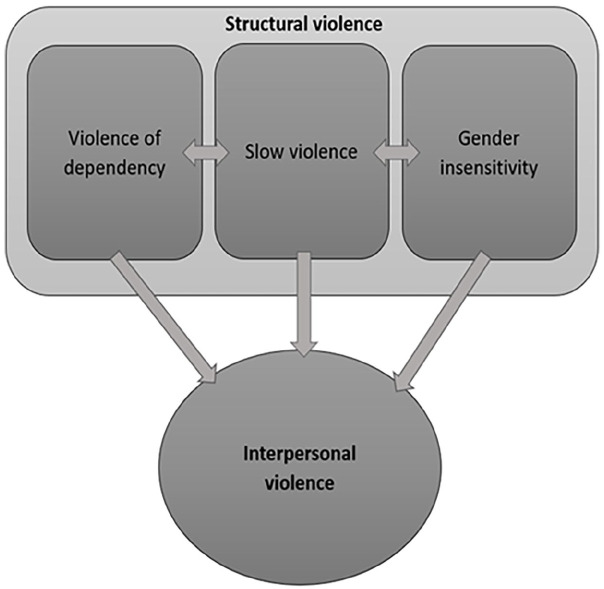
Three forms of structural violence support the harms of interpersonal violence.

The term “structural violence,” introduced by Galtung (1969), delineates the invisible structures of inequality and injustice that cause harm to individuals through an indirect and insidious process, embedded in long-standing “ubiquitous social structures” normalized by stable institutions (Gilligan, 1996). The “simultaneous” consideration of intersectional vectors of oppression, including racism, war, poverty, and gender inequality, amounts to “a political economy of brutality.” In other words, it is a single axis that defines an increased risk for extreme human suffering (Farmer, 1996: 274). Structural violence exists alongside, and interacts with, the continuum of violence (Roupetz et al., 2020). The harms occasioned by structural violence are preventable and not committed by an individual actor. Rather producing harm is produced via the inequality that is built into structures, herein immigration and asylum systems. While a much-cited theory, structural violence has rarely been explored empirically (Figure 1) (Hamed et al., 2020).

This article examines how structural violence generates direct harms *and* facilitates interpersonal violence. The “little routines and enactments of violence” practiced normatively in institutional and private settings (Scheper-Hughes, 1995, p. 143; Scheper-Hughes, 2004) are rendered invisible; they are widely perceived as the status quo, but experienced as assaults on dignity (Price, 2012), leading to erosion of the individual’s agency. Through repetition and reiteration, violence becomes invisible and normalized (Hamed et al., 2020). In our analysis, we argue that immigration and asylum systems exacerbate existing vulnerabilities for SGBV survivors, and we identify three types of structural violence: the intimate violence of dependency; slow violence; and the violence of gender-insensitivity. We construct a heuristic framing of structural violence in conversation with scholarship on structural violence, forced migration, and SGBV. We use our data to show how these violences exacerbate risks of, and exposure to, SGBV, resulting in gendered harms (Canning, 2017).

### The Intimate Violence of Dependency

Asylum and immigration systems often reify women’s and children’s dependencies on men by rendering independent support inaccessible and creating conditions for abuse. Normative assumptions around family, heterosexual reproduction, and marriage pervade asylum and immigration systems (Giametta, 2017), determining residency rights and access to services. Decades of domestic violence research have shown that the abuse of intimacy to ensure power and control over a domestic partner is injurious: physically, psychologically, and financially. Such abuse is a feature of spousal and other intimate relationships during asylum-seeking. Women and children often remain in conflict or camps (Cockburn, 2014), while men are more likely to migrate to seek asylum and, after gaining status, seek reunion or marriage (Pertek &Phillimore, 2022). Partners joining someone with refugee status^1^ are granted a spouse visa, becoming dependent on their spouse, despite being forced migrants themselves in need of protection. Even when families arrive together, a male household head is often allocated “lead asylum applicant” status with the wife considered dependent. Spouses must remain in a residential married relationship for a probationary period of between 2 and 5 years. Some countries even restrict the dependent spouse’s recourse to public funds throughout these periods, including access to domestic violence refuges, while preventing access to employment, which creates financial dependency (Shabbar, 2012).

Spousal visas and secondary asylum statuses constitute a form of structural violence; a technology of bordering disrupting boundaries of public and private lives (Innes and Steele, 2015; Turner & Espinoza, 2021), creating an enforced dependency that offers “a powerful tool of oppression” (Mirza, 2016, p. 592). Provisions such as the Domestic Violence Rule (U.K.) and its equivalents, which could enable individuals to remain after leaving an abusive relationship, offer limited protection as they are not publicized; little information is translated into non-native languages; and the burden of proof is excessive (Voolma, 2018). Spouses leaving abusive relationships will be returned to unsafe situations where they may face persecution or family violence (Papoutsi et al., 2022). Thus, the immigration conditions to which spouses are subject operate as a form of structural violence by denying safe exit from IPV and blocking access to services and conditions that could enable independence. Canning (2020) refers to such IPV as corrosive control, noting that harm is inflicted by state structures of control. Those arriving on spouse visas will avoid engaging in asylum systems, *if* they are able to wait out their probation periods *or* successfully apply for a domestic violence concession. Those seeking asylum face gender-insensitivity within asylum processes.

### The Violence of Gender-Insensitivity

Displacement experiences differ for men and women, with gendered power imbalances playing out across the continuum of violence. Women are more likely to experience SGBV (Cockburn, 2004), and men are more likely to be persecuted for their politics (Baillot et al., 2014). Asylum determination regimes have been described as “gender-blinkered” (Poynting and Briskman, 2020, p. 5), with the normative asylum seeker being male. A presumption of “bad faith” (Poole & Rafanell, 2018) fosters a culture of disbelief which tends to discredit the validity of asylum claims (Souter, 2011). This is particularly important because credibility is a key criterion in asylum claims processing, with excessive demands for narrative detail and “proof” (Schuster, 2020). Even though it is widely acknowledged that SGBV is widespread among women forced migrants and constitutes a gendered tool of persecution in forced migration, many non-governmental organisations (NGOs) express concerns about the mishandling of survivors’ cases (Baillot et al., 2014). Asylum interviews are described as cruel, with interview techniques causing considerable distress (Kjelsvik, 2014). SGBV claims are viewed as “risky” as they are hard to prove and SGBV is not viewed as “persecution” (Baillot et al., 2014).

The combative environment in which women must disclose SGBV produces fear of engagement, thereby preventing disclosure (Goodson et al., 2020). Further, the shame and trauma associated with SGBV can prevent women from disclosing until it is a last resort (Papoutsi et al., 2022), with caseworkers failing to acknowledge that disclosure is difficult (Griffiths, 2021). Scholars point out that such adversarial asylum systems, with their poor decision making, and the questioning of credibility are detrimental to individual’s health because their lived experience is denied (Baillot et al., 2014; Beneduce, 2015). Thus, the failure to be sensitive to the gendered nature of forced migration is a form of structural violence that can potentially re-traumatize survivors, and result in rejection and abandonment. Asylum-seeking survivors and spousal migrants with no recourse to public funds are also subject to the slow violence inherent within asylum systems.

### Slow Violence

The deliberate deprivation of resources within asylum support systems have been conceptualized as slow violence, which is incremental, occurring across time and place and intended to disenfranchise (Mayblin et al., 2020). Those in U.K. asylum support survive on an extremely low income, relying on the cheapest food aid from charities (Mayblin et al., 2020). Forced migrants are placed in housing often considered unfit for human habitation (Bakker et al., 2016), and/or are moved around the asylum estate where a politics of discomfort undermines their safety (Darling, 2011). The abandonment of forced migrants is another form of slow violence, with the intentional deprivation of those residing in appalling conditions in the Calais camp, “the Jungle” amounting to structurally sanctioned dehumanization through withholding food, medicine, and sanitation (Davies et al., 2017). Papoutsi (2021, p. 3) has demonstrated that in camps, abandonment functions by design, “through daily routines, periodical events and cycles of waiting.”

The slow violence of abandonment is also evident in the treatment of those whose asylum claims are rejected or those who leave a spouse upon whom they are dependent. In the United Kingdom and Sweden, no recourse to public funds rules exclude most spousal migrants and rejected asylum seekers from welfare and work. Withdrawal of support results in destitution and homelessness when someone is between asylum appeals, and/or hiding from an abuser, and potentially dependent on others’ generosity (Alpes et al., 2017). Such slow violence may generate particular harms for SGBV survivors who find living with strangers re-traumatizing, are fearful of returning to further SGBV, and subject to increased risks of violence when destitute (Singer, 2012). The three forms of structural violence outlined so far create harms independently for SGBV forced migrant survivors, but can also occur simultaneously, thereby compounding harms.

### Context

This analysis draws on data from the (SEREDA) project, which explored the experiences of SGBV survivors across a geographical continuum of violence from pre-displacement to refuge. We draw on interviews with women SGBV survivors living in Australia, Sweden, and the United Kingdom, subjected to immigration or asylum policy. Countries were selected because of their diverse histories of forced migration arrival and policy, which enabled us to identify commonalities and differences using comparison as a parallel demonstration of theory, wherein findings have enhanced validity if appearing across multiple contexts (Skocpol & Somers, 1980). We focus on those with spouse visas using “marriage for refuge” and asylum seekers. Australian interviews took place online during the pandemic and multiple lockdowns. To ensure all respondents were safe we needed to know their living arrangements so we interviewed 16 forced migrants who had previously participated in a project on family violence and who either arrived as marriage migrants or were tied into their marriage under the terms of their resettlement. We were unable to access asylum seekers in Australia. Below, we outline the policies to which survivors were subject between 2018 and 2020 when primary data collection took place (Table 1).

**Table 1.** Conditions to Which Spousal Migrants and Asylum Seekers Are Subjected.

Country	Spousal Visas or Dependent Permit	Asylum Conditions
Australia	Resettlement dependent on family status^a^ Must reside with partner for two years^b^ Generally ineligible for social security with some exceptions (case to case)^c^ Can apply for permanent residency status through Family Violence Provisions in the Migration Regulation (no waiting period)	Not included in study
Sweden	Must reside with partner for two years^d^ Extended residence permits can be granted if the relationship has ended due to violence^e^ Documentary evidence of violence needed to prove relationship ended due to abuse.	Disclosure of IPV or SGBV when asylum first claimed is expected and later disclosure has to be explained High levels of proof of fear/occurrence of violence required by the migration board^f^ Rejected AS NRPF and likely to be semi-detained in pre-deportation accommodation
U.K.^g^	Must reside with partner for two or five years. NRPF (including to use IPV refuges). Application to remain under Migrant Victims of Domestic Abuse Concession requires documentary evidence of abuse. Fee payable depending on financial circumstances. No recourse to piblic funds (NFPF) while application is processed.	If “coupled” lead asylum case likely to be male. Two interviews required alongside a witness statement and evidence (i.e., photos, medical reports). Unclear when SGBV should be disclosed but failure to do so consistency undermines credibility Eviction and NRPF if claim rejected, possibly followed by detention and deportation. Rejected AS not permitted to work.

*Note.* NRPF = no recourse to public funds; SGBV = sexual and gender-based violence; IPV = intimate partner violence.

a Key facts on the conflict in Syria and Iraq. Key facts on the conflict in Syria and Iraq Access 2/2/25.

b Hoogenraad (2021).

c inTouch Multicultural Center Against Family Violence, Immigration: Resource for working with women on temporary visas. Undated tip sheet covering period of interviews.

d Voolma (2018).

e https://www.migrationsverket.se/English/Private-individuals/Moving-to-someone-in-Sweden/Extending-a-permit/If-the-relationship-ends.html.

f https://www.migrationsverket.se/English/Private-individuals/Moving-to-someone-in-Sweden/Extending-a-permit/If-the-relationship-ends.html.

g U.K. Government (2024).

## Methods

Some 102 interviews were conducted with women forced migrants to explore their experiences and impact of SGBV. We partnered with NGOs who provided a range of services, including around SGBV, to forced migrants. Partners helped us design research tools and develop the ethics protocol. They approached current or former service users (SUs) who were not in therapy and described the study. If SUs wished to participate, partners shared the information and consent forms in the appropriate language and requested to share contact information with a bilingual researcher. Researchers contacted respondents via e-mail, text or WhatsApp and arranged to talk. The study was again described before verbal consent agreed. A date was set for the interview in a location of the women’s choice, consent was requested, and forms were signed by the researcher and the participant. The participant was told they could stop at any time, end the interview, and withdraw their participation and their data up to 30 days after the interview was completed. Nobody chose to do this.

We also approached other NGOs with whom we had a working relationship and utilized snowballing sampling, asking respondents if they knew of others who might wish to talk. Some interviewees were identified by respondents and others via our own networks, social media appeals, and following the distribution of fliers. Interviews were sought with survivors with different immigration statuses, genders, and sexual orientations. Participants mainly originated from the Southwest Asian and North African (SWANA) or Sub-Saharan Africa (SSA) regions, which were selected because they represented the largest groups of forced migrants in the countries but had experienced different forced migration journeys. Many SWANA respondents arrived in the United Kingdom and Sweden after traveling on foot and by boat during 2015 to 2016. The SSA respondents often endured journeys of several years. Most respondents were women, reflecting the general profile of SGBV survivors (Johnson et al., 2008).

In this article, we focus only on women. Overall, some 55 respondents originated in the SWANA region, 44 from SSA, and 3 from other countries (see Table 2). There were 29 asylum seekers, 43 refugees, 15 rejected asylum seekers, and 13 individuals arriving on spouse visas. Some 73 women identified as heterosexual, and 3 as lesbian (24 participants did not disclose sexuality). Fifty-nine participants were between 30 and 50 years old, with ages ranging from early 20s up to 70 years old. Some 34 were single, 27 partnered, and 40 divorced, separated, or widowed. More interviews were completed in the United Kingdom because additional funds enabled expansion of our work to include forced migrants from SSA. Interviews were semi-structured and conducted in interviewees’ chosen language either by bilingual researchers or with a trusted interpreter. Interviews ranged from 30 to 90 min, mostly digitally recorded, with notes when permission to record was refused. Interviews covered topics such as experiences of violence across the lifespan, how SGBV is discussed within communities, help received, coping strategies, and rebuilding lives in refuge. Here, we focus on structural violence, which emerged as a key theme. We do not herein examine help received, beyond noting that some received no assistance but those findings can be viewed on the X website.^2^

**Table 2.** Summary of Sample.

Country	Australia	Sweden	U.K.	Total
Immigration status				
Asylum-seeker	0	3	26	29
Rejected asylum-seeker	0	0	15	15
Refugee	11^a^	13	19	32
Spouse	5	1	7	24
Not known	0	0	2	2
Sexuality				
Heterosexual	16	15	42	73
Homosexual	0	0	3	3
Not known	0	1	24	25
Region				
SWANA	16	17	22	55
SSA	0	0	44	44
Other	0	0	3	3
Total	16	17	69	102

*Note.* SSA = Sub-Saharan Africa; SWANA = Southwest Asian and North African.

a Arrived under Syria and Iraq resettlement program which prioritized families.

Audio files were transcribed, anonymized, and translated into English. The transcriptions and notes were coded in NVivo, using codes developed collaboratively across the project team, with Author 1 checking validity. When developing themes for analysis, we observed wide-ranging harms occasioned by immigration and asylum systems. We created a code, “policy, justice and legislation” and sub-codes (references to governance; barriers to justice, experiences of mechanisms, effects of immigration/asylum policy and legal consciousness), and a further code “structural violence” (violence caused by state apparatus and apparatus exacerbates SGBV), under which the harms generated by state structures were coded.

Ethical approval was gained from each institution’s ethics boards. The whole team was trained in-person in trauma and gender-sensitive interviewing by Author X, an expert on this topic. We followed a protocol setting out the procedures to follow if the interviewee or interviewer became distressed, for trauma-sensitive interviewing, and which listed organizations interviewers could refer respondents to if they became distressed, including help from partner organizations. Interviewers were asked to restrict the number of interviews they undertook daily and were given optional access to psychological support. Each transcript was anonymized and allocated a pseudonym that reflected respondents’ countries of origin. Interviews and pseudonyms were stored at the University of Birmingham secure data facility with only team members able to access data. After data were analyzed Phillimore and Goodson ran workshops with NGOs and some survivors to gain feedback on findings and identify priorities for policy work. The names used in this article are pseudonyms.

## Findings

Below we set out the ways in which forced migrants were subjected to the three forms of structural violence and how these violences exposed them to interpersonal violence and in some cases further SGBV.

### Intimate Violence of Dependency

Women respondents discussed experiences of SGBV when their residence was tied to their refugee spouse via visa conditions. They described requirements to maintain their marital relationship and how abusive partners used their power to assert control and sometimes inflict cruelty. The inequities in the spousal migrant relationship, imposed by the state, meant that survivors often experienced precarious existences, sometimes subject to multiple forms of violence, having no option, other than deportation, but to remain in their marital relationship. Partners appeared to know they could act with impunity.

Some participants recalled the different tactics their abusive husbands would use to get their demands met. Lilan, a Kurdish spousal migrant in the United Kingdom described being subject to coercive control by her husband, enabled by corrosive state control:

He never let me to go out at all, then he was working at a factory, he go to work, and then lock the door and he work, and then when he came like that it’s 9 hours he was at work, and then I was locked at home all day, even I couldn’t open the curtains, he closed the curtains.

When threatened with divorce and return to her family where she feared honor-based violence, Lilan did what her husband asked. When she left after he tried to choke her, she could not prove how long she had been a resident in the United Kingdom because he destroyed her documents. She was detained prior to attempts at deportation.

Several women recollected how their husbands’ decisions to end the relationship, sometimes followed by a call to immigration authorities, resulted in their detention. Hamia, from Algeria, was forced to marry a British man who beat her, then used her immigration status to get her imprisoned “I live with him about eight months then he called the Home Office, they put me in detention. Abide, from Jordan also found herself detained in the United Kingdom after leaving her abusive husband ‘I ended up spending 7 months in detention! I wasn’t allowed to use Skype to talk to my father. They were treating women really badly in the detention.’”

Women in relationships where their male partner was the lead asylum applicant also experienced dependency. When Maree’s husband, from Zimbabwe, gained refugee status, the power dynamics in their relationship changed. She said he became violent and refused to support her case, despite the presence of their child:

I didn’t have papers and he was supposed to sign the papers for me, as his beneficiary but he refused. So, then there I was, and he wanted me out of the house. According to him this is my house you are not welcome here; you can’t live in this house. I didn’t have anywhere else, and our son was living in that house.

Women were sometimes tricked into “marrying” and starting a new life in the United Kingdom and Australia, only to discover that their husband used their dependent status to force them to work as cleaners or sex workers. Women were threatened with divorce and detention if they did not hand over their money or perform their work. Several women in the United Kingdom explained how they learned from NGOs that they had been trafficked. Living for years in controlling situations, they were unaware that, rather than breaking the law, they were victims of crime and could apply for asylum and protection.

Many respondents detailed how the asylum and immigration system made them financially dependent on their husbands. We heard of women and children going hungry because naturalized partners controlled the finances and would not buy food. In Australia, welfare benefits were paid directly to the husbands of women on spousal visas. Alenya, a marriage migrant from Lebanon described how, even after leaving her husband, she could not access welfare because “[h]e used to take the money from Centrelink and not spend it on his son. At the time, they couldn’t transfer the money to my name because I didn’t have permanent residency.”

Several asylum applicants in Sweden, all with their own welfare service support cards, spoke of how they had to hand their cards to their husbands. Halima from Syria explained “I have [the card] but to be honest it goes all to him; he takes it all and doesn’t leave anything with me. He always likes to prove that he’s the man.”

Women expressed fears that if they left their partners, they and their children would be destitute, which is reflective of the reasons why women in the wider population cannot leave abusers (Heron & Eisma, 2021). Some respondents in each country said they remained because the husbands threatened to remove their children if they ended the relationship or if “authorities” heard about the abuse. Interviews with service providers confirmed that losing the children to social services or a partner was a common fear, as expressed by Mena, the spouse of an Iraqi asylum seeker in Sweden: “So, then he said, ‘Leave your son, leave Youssef, but you go. I will give him to a Swedish family who wants to adopt him.’” Arram, also from Iraq, spoke no English and relied on her husband to explain life in Australia. She reported being denied access to basic information, with her husband telling her she was too stupid to remain in the country. He warned her to behave, or she would lose her children: “The only thing he used to tell me about Australia was that if you misbehaved in public, they take your kids away from you and put you in prison.”

However, we did encounter examples in Sweden and Australia wherein dependent spouses, refugees, or asylum seekers gained independent leave to remain and were no longer subject to immigration and asylum systems. In that instance, they felt empowered to leave abusive husbands, which reinforces the claim that these systems had been subjecting women to structural violence. In Australia, gaining citizenship marked the moment Bariqa, a marriage migrant from Lebanon, felt she could safely divorce. She narrated leaving her husband after passing her test:

Eight and a half years I’ve been waiting for my citizenship to come through. After my citizenship came through, I said to my mum “that’s it. This man has passed his expiry date, it’s over.”

Women could leave relationships after being informed by NGOs of their rights, often following an IPV referral, or because their length of residence entitled them to remain and access state resources, removing the threat of destitution (Heron & Eisma, 2021). In Sweden, the existence of a strong public sector working against IPV was an important factor. For example, Dunia, from Iraq, was told by her violent husband that no one would help her. She was terrified of the prospect of returning to the relationship if unable to achieve financial independence. But, after being referred to the Swedish Social Services by an NGO, she reported being told: “we will not stop supporting you until you become capable of standing on your own,” which gave her the confidence to finally leave.

The precarity and liminality produced by long and dehumanizing periods of dependence, living in fear of being deported and bringing shame to their families had a psychological cost, as women were made to be constantly “at the border” (Sampson, 2013). They endured interpersonal, physical, and sexual violence at the hands of their abusers, believing they were unable to leave, and experienced psychological distress, with three respondents hospitalized following a breakdown. Long after the end of relationships, women reported continued psychological harm. Thus, the structural violence of the immigration and asylum systems created violent intimacy, underpinning and enabling interpersonal violence and health harms. Some were forced to go through separate asylum processes when they separated from the primary applicant, while others who sought asylum independently also faced these systems, which were largely insensitive to gender.

### Violence of Gender-Insensitivity

Forced migrant SGBV survivors must navigate asylum systems without knowing how those systems work. The systemic gender-insensitivity means that the different types of persecution experienced by men, women, and LGBTQI people are not acknowledged, as the tacit working assumption is that the standard asylum seeker is male (Le Bellec, 2021). SGBV is not perceived as a legitimate and sole basis for claiming protection (Baillot et al., 2014), while women’s caring responsibilities, or the shame and secondary trauma associated with SGBV disclosure (Papoutsi et al., 2022), is not accounted for. Respondents explained how the legitimacy of their claims, including their SGBV narratives, was challenged by officials. Respondents recalled lengthy interviews wherein hundreds of questions were asked over many hours while women were separated from their children, often for the first time. Eden, a single mother of three from Eritrea seeking asylum in the United Kingdom, recalled feeling “treated like an animal.” She felt pressured to give the answers that the interviewer wanted to return to her children. She was asked over 500 questions, feeling increasingly stressed:

I don’t know what the right thing, I learn, I am like that, I have to be free quick, to have interview to search baby, I don’t want to lose time, that time. So, what he want I do, he say I told you, this I told you, you know, he make me like that, when he tell me like that, I become stressed.

Many respondents in the United Kingdom, especially from SSA, described asylum interviewers questioning the truthfulness of their accounts of SGBV. Binta, from Sierra Leone, was interviewed at length. Having disclosed extremely sensitive information, which she had shared with very few people, she was threatened with detention: “she was shouting ‘oh you’re lying we are going to put you in detention!.’” Respondents recalled interviews bringing back traumatic experiences as they broke down but were told to continue, then were sent away without support or counseling. Re-traumatization is a frequent occurrence when disclosing SGBV, even in supportive settings (Mezey et al., 2005). Some spoke of having flashbacks during parts of the interview. Olanrewaju, from Nigeria and seeking refuge in the United Kingdom, described the distress she felt:

For two days I couldn’t eat even when I go home despite all those things. I think you know at those times not only asking, but they should help, help to cope with the trauma you know of bringing it back.

Respondents awaiting a decision experienced a sense of hopelessness, believing, in the absence of any explanation of how things worked, that decisions depended on luck or on whether the caseworker liked you.

Interviews were also problematic because of the quality and nature of the interpretation. In some cases, interpreters did not speak the same language as respondents. Some survivors were unable to disclose SGBV experiences in the presence of a male interpreter or someone from their community due to the fear of shame for them and their family (Sullivan et al., 2023). Souleymane, from Senegal, recalls her concerns:

Sometimes my English is not good for them, they get interpreter, any I don’t want in front interpreter, I told you, my country people, not like this, when they talk, they talk, when he see me, this is here, this is everywhere.

Aggressive interview techniques and practices exacerbated feelings of shame, making respondents feel criminalized. Ahu, a Kurdish woman seeking asylum in Sweden, told her interviewer about the violence she encountered at the hands of the Turkish police, which had left permanent injuries. She was astounded that in her interview of over 4 hr she was repeatedly asked if she or her family were terrorists, with the interviewer apparently uninterested in the evidence she provided of her persecution over her humanitarian work:

They ask if I am a member of any terrorist organization, and why my humanitarian work has perceived as membership to a terrorist organization. Is any member of your family affiliated to a terrorist organization? Have you met members of a terrorist organization? Did they ever contact you after you got here?

Overall, respondents in Sweden felt they were reasonably well treated in interviews, but in the United Kingdom, women reported being humiliated and criminalized when they reported at Police Stations or went to court for a hearing. Zaineb, a Jordanian asylum seeker, told us:

Then, the Home Office is criminalising me since 2012! They keep searching my clothes, I used to be taken to the court with police all around me with handcuffs! All the humiliation from my ex-husband, everyone, and the Home Office . . . I go to sign at the Home Office every two weeks, then every month. It was like a nightmare. I used to go in terrified that they might put me back to detention.

Several respondents talked about the dehumanizing way they were treated. Such *othering* was experienced as an intensifying sense of unbelonging. Many respondents had previously been sexually assaulted or physically beaten by authority figures, especially police and prison guards. Having to report to Police Stations and being detained was terrifying, as explained by Abina, an asylum seeker originating in Ghana who had been trafficked and imprisoned at various points on her journey, including in the United Kingdom: “It was really terrible to me, like to me when I was at the detention, I was thinking I didn’t committed any offense, why would I be locked up?”

Structural violence for some women was in the form of lack, or withdrawal, of care, which Davies et al. (2017) refer to as a state of abandonment. We heard from women in the United Kingdom and Sweden that they were denied access to essential medication and had their access to medical care rationed, which was particularly harmful because their injuries resulting from SGBV needed consistent treatment. Contact with clinicians was treated as a bureaucratic matter and not a setting wherein women felt comfortable to disclose SGBV-related health complications. Olanrewaju, a (now) refugee from Nigeria, recalled a lengthy story of untreated blood loss:

It took them weeks for them to book an appointment for me to check my blood, and when they checked my blood, they discover that I had very low haemoglobin, dangerously low.

Nonetheless, Olanrewaju was expected to attend lengthy asylum interviews and only received proper treatment once released from detention. She explained that she felt she was being “left to die.” Olanrewaju was prescribed antidepressants for the trauma experienced in detention, which was totally preventable.

In Sweden, Ceren recalled her treatment on arrival as an asylum seeker from Turkey. After disclosing SGBV, she was sent for repeated medical examinations, which forced her to relive dehumanizing experiences (Mezey et al., 2005) with consequent re-traumatization because the examinations were to assess the veracity of her claim, rather than for therapeutic reasons:

Although I told them the violence that had happened to me at first examination, I was asked again later somewhere else. Now I think that they will ask me this question and I will experience this trauma over and over again every time I go to the hospital.

Layan from Syria, living in Sweden, described poor access to medical care in camps. Despite being unwell, she was not given access to care until another asylum seeker helped her to communicate in English: “My medical condition at this point was very deteriorated and I couldn’t tell them I was pregnant, so I didn’t receive any medical assistance.” Alia described how poor conditions, including overflowing toilets and terrible food, led to her daughter becoming so sick that she had to be taken to the hospital:

My daughter went through a lot here, she was taken by the ambulances several times; she would either have a fever or be allergic to somethings. I would cry all day for the first six months, there wasn’t a day that passed where I didn’t cry.

Inhumane asylum interviews that were insensitive to the respondents’ gendered trauma, encounters with male authority figures, and detention without having committed a crime were coupled with the failure to attend to migrants’ physical and psychological needs. Rather than offering the support and protection survivors needed for their recovery from SGBV, immigration and asylum systems re-traumatized respondents and did not address their health problems, causing harm that some respondents said endured for many years even post-asylum. This was often compounded by slow violence.

### Slow Violence

In this section, we look at the everyday slow violence (Mayblin et al., 2020) produced by the asylum system. A common theme was unsafe and unstable accommodation allocated on a no-choice basis. Earlier work has reflected on the tendency to house asylum seekers in the cheapest housing available, typically located in the most deprived areas (Phillimore & Goodson, 2006). Housing conditions, including leaking roofs, unsafe stairwells, mold on walls, and broken heating and hot water systems, were described by Sade, from Nigeria who had been moved around for years when she was seeking asylum:

I’ve lived in all sorts of accommodation really very, very bad accommodation from, and these accommodations given by the Home Office. Most of the accommodation is not suitable for animals, much more human beings.

In all countries, we heard reports of unfit and unsanitary housing. After arriving in Australia from Syria, Sosamma was told that dirt and insect infestation was normal, and she would have to learn to live with it:

I don’t think anyone has cleaned the room for a year maybe. And I got bitten, like a huge bite and it got really big, from the mattress that I was sleeping on.

Women said that experiencing such conditions day-to-day made them feel they were viewed as “animals” unworthy of better.

Some participants in the United Kingdom and Sweden discussed placements in mixed gender housing and being repeatedly harassed by other residents and staff, with several reporting attempted rapes. Women lived in fear because there were no single gender facilities or security measures to keep them safe. Such housing arrangements made them feel particularly vulnerable, given the violations they had already experienced. As Faiza, a Palestinian asylum seeker in the United Kingdom, recounts:

The doors of the toilets and showers don’t have locks. I have to wait and make sure people are asleep in my floor so I could use the toilet. I keep the door pushed with my hand while using it. . .. Why aren’t there locks?!

Mandatory movement to new accommodation, sometimes within the same city but often many miles away, disrupted the formation of networks with peers, NGOs, health providers, and schools, all of which were crucial to migrants’ recovery from trauma. This type of disruption, sometimes at very short notice, was disempowering and upset the routine that survivors had established to develop a sense of normality; a situation that Darling (2011) argues is intended to cause discomfort and disconnection. Maria, a Guinean asylum seeker in the United Kingdom, explained how she was moved several times and once given just 5 minutes to leave with no offer of help to remove her possessions despite having to care of a baby: “You don’t have any help, it was really, really stressful, I have to take two taxis with the baby and the pram, I got all those stuff to another temporary accommodation.”

Even though she now had refugee status in the United Kingdom, Maria struggled to feel at home because she had been moved around so much over the previous 8 years. In Sweden and the United Kingdom, asylum seekers were unable to choose to move unless they had permanent residency, or someone was prepared to house them. Several respondents struggled with the cold weather and darkness in Northern Sweden, not having the right clothing to cope with the conditions. Aala from Afghanistan was terrified “it would be dangerous for my children” so moved into a single room provided by a friend in the South “in order to survive that situation.” Kirchner and Pääkkölä (2020) found health risks associated with cold temperature exposure of refugees.

When placed in detention, hostels, and hotels, forced migrants must eat the food provided at given times or go hungry. Sometimes the food was inadequate “they provided only one meal per day” (Afghan, asylum seeker, Sweden). We heard how the poor-quality food made respondents physically sick. Food available in asylum accommodation differed considerably from the diet respondents were accustomed to, consisting of fried refined food that caused stomach problems, a problem identified elsewhere (Fu et al., 2022), and argued to operate as a form of control in asylum regimes (Amir & Barak-Bianco, 2019). Esin, a rejected asylum seeker originating in Afghanistan, reflected on her experience in Sweden, and described how, despite the food making her children sick, no alternative provision was offered:

. . .We said to the Migration Board that our children were sick—“this is not place for people.” And they said “these are the places that you can have because you have been refused. Either you go back to Afghanistan, or you stay here!”

Asylum seekers living independently also faced daily struggles because of below poverty-line levels of asylum support (Mayblin et al., 2020). Survival for those in food poverty depended on finding the cheapest goods and sharing with others. In Sweden and the United Kingdom, the payment card given in lieu of cash could only be used in certain stores, making cheaper products in other shops and/or online unobtainable. In Sweden, respondents said they were ashamed of being asylum seekers, and described facing racism and discrimination because the payment system made them visible. As Rose, who finally gained refugee status in the United Kingdom, explains, eventually such treatment undermined her self-confidence, making it difficult to rebuild her life.

Life is a struggle. And that discrimination really reduces you, it strips you of your self-confidence, you become someone else, because you are not free. So, I would say this also is in a way increased the abuse.

Some respondents spoke of how they were asked to prove their identity when shopping with the card, even though shopkeepers had no right to ask. Such practices reinforced the sense of shame and unbelonging (MasGiralt, 2020) as the structural violence of asylum systems played out in everyday life and spaces. Bureaucratic practices or mistakes could lead to the cessation of support without notice, leaving survivors destitute, dependent on others, hungry, and at risk of further exploitation. In the United Kingdom, one woman described how she had been unable to withdraw her weekly payment because she had been in labor, so “they take the money back.” In Australia, problems transferring refugees to welfare support left them without food.

Individuals who gained refugee status in the United Kingdom and Australia continued to struggle as they found that welfare benefits did not cover the costs of housing and food. Sossie from Lebanon struggled to survive on meager welfare payments after leaving her abusive husband, having to choose between food and rent: “I used to save the money for rent, and not spend it on food.” While this is the case for all people on low incomes, refugees felt particularly vulnerable because they had no material possessions or family who could help them financially. Bagiyeh, a resettled refugee from Iraq, explained her frustration:

You know, I often dream about one day coming face to face with Scott Morrison [then Australian Prime Minister] and tell him that you need to make a distinction between the people who have family and relatives here, and people to support them. We’re not all the same.

In the United Kingdom and Sweden, respondents were made destitute and homeless when their asylum claims were rejected. Some women respondents said they had to enter risky relationships to secure shelter, sometimes returning to abusive relationships. Research has also shown that limited financial resources is a factor in domestic violence survivors’ return to abusive relationships (Griffing et al., 2002). Maria from Guinea, who claimed asylum in the United Kingdom after being abused by her husband when on a spouse visa, described how she lived after her claim was rejected:

I was sleeping from one sofa to another just to make ends meet. I’ve lived with different, different men, they use me when they finished, they told me to leave the house. [. . .] That go on since end of 2005 until 2010.

Still without refugee status when interviewed 13 years later, Maria, like many other women we spoke to, relied on friends and charities for survival as she was abandoned by the state.

Unsafe housing, poverty, and cycles of destitution made everyday life a struggle for respondents, adding a further layer of trauma to earlier experiences of violence, the re-traumatization caused by gender-insensitive treatment in asylum systems and SGBV at the hands of husbands or in conflict. Without access to work, stable housing and under constant threat of homelessness and hunger, the reality for respondents was a life of uncertainty. Post-migration trauma, in the form of extreme impoverishment, poor living conditions and uncertainty about the future (Grace et al., 2018) has been shown to generate trauma at least equivalent to that experienced in conflict and flight (Steel et al., 2017) and to have long-term effects on physical and mental health (Phillimore and Cheung, 2021). These conditions do not occur accidentally. Such conditions are implemented by states to deter people from seeking asylum despite the lack of evidence that such deterrents are effective (Di Iasio and Wahba, 2023). As we show here, these measures combine to undermine the protection of SGBV survivors, placing them in unsafe situations, exposing them to violence and further harms.

## Discussion

In this article, we detailed some of the ways that immigration and asylum systems are fraught with structural violence and addressed the question of how this violence impacts the lives of women forced migrant survivors of SGBV. We show how forced migrant women survivors, deemed by UNHCR to be among the most vulnerable of displaced people, and entitled to special protections, experience “multi-axial models of suffering” (Farmer, 1996). We provide an important operationalization of the theory of structural violence (Hamed et al., 2020), building on Canning’s (2017) arguments about the gendered harms introduced by U.K. immigration and asylum systems, to offer a new analytical framework. Drawing on the work of Galtung (1969) and others, our framework contributes to the growing literature on violence in immigration and asylum regimes in three ways. We offer a gender-sensitive perspective that unveils the traumas and harms that women endure while seeking refuge. We also dissect this systematically and examine how the different forms of structural violence intersect. We demonstrate how the “political economy of brutality” (Farmer, 1996, p. 274) works in everyday contexts across three countries, sabotaging women’s prospects for recovery and integration. Finally, our paper dialogues with the literature on the continuum of violence (Roupetz et al., 2020), offering empirical depth to this concept and showing how the continuum extends to the private sphere (i.e., dependency) and to refuge. This is important because dependency extends border violence into intimate spaces, into the family sphere, demonstrating how borders and patriarchy intersect, compounding gendered harms.

Our work shows how women are routinely failed and further vulnerabilized by the systems meant to protect them, leaving survivors faced with interpersonal violence, thereby reinforcing claims by Galtung (1969) and others (Roupetz et al., 2020) that structural violence generates other forms of violence. We show how these invisibilized normative routines and enactments of violence settings (Scheper-Hughes, 1995) result in multiple forms of interpersonal violence, including further exposure to SGBV. We demonstrate how the intimate violence of dependency is imbued with and reinforced by normative heteropatriarchal assumptions, and embeds profoundly unequal power relations into marital and intimate relationships. The disempowering effect of such policies is shown by migrant women staying in abusive settings when subject to immigration restrictions, and leaving when these are removed. Violent dependency instills the precarity of constantly being at the border (Sampson, 2013) with abusive partners operating as border guards. Those who leave are criminalized and subject to detention, and at risk of honor-based violence or facing gender-insensitivity within asylum systems.

The violence of gender-insensitivity is evident not only in, first and foremost, the failure to recognize SGBV as a gendered tool of persecution but also in the failure to understand the challenges of disclosure and the consequences of aggressive and combative questioning. Gender-insensitivity generates re-traumatization in the process of asylum claims determination (Kjelsvik, 2014). We heard accounts of cruelty with: interviews sometimes undertaken when women were unwell; lengthy interviews without breaks; separation from children; and no access to post-interview trauma counseling (Steele & Brent, 2013). Gender-insensitive interviews meant that no attention was paid to the shame and stigma associated with SGBV disclosure (Papoutsi et al., 2022) nor to the need for confidentiality and discretion. In contravention of guidelines on interviewing survivors of sexual assault (Lonsway et al., 2022), women were challenged about the validity of their accounts and constantly asked to reiterate experiences of the violence. Häkli and Kallio (2021) examine embodied encounters in asylum interviews, differentiating between objective bodies and subjective persons, to argue that the refugee body can be politicized in attempts to control and disempower, wherein individuals are treated as a faceless problem. Certainly, women spoke of being dehumanized as objective bodies or “animals” in interviews. Gender-insensitive treatment exacerbated psychological trauma, sometimes continuing years after the original asylum interviews. Gender-insensitive statutory asylum determination structures represent “a systemically cruel state disposition toward asylum seekers as part of a regime of deterrence from seeking asylum” (Poynting & Briskman, 2020, p. 3) that generate enduring harms. A small sign that statutory processes can improve their gender sensitivity is the introduction of “Leave this page now!” buttons on Swedish migration board web pages,^3^ along with advice about how to “hide your visit” to protect migrant women seeking to escape abusive partners. The appearance of such minimal concessions to the risks faced by forced migrants highlights the extent of work needed.

We argue that some of the conditions to which dependent women are subject, for example, restricted access to food, are a form of slow violence and can compound gender insensitivity and violent dependency. Poor living conditions make survivors physically and psychologically unwell, while they are often placed in unsafe situations and separated from vital support networks when in state-allocated housing (Mayblin et al., 2020). Everyday experiences of poor-quality food, forced mealtimes, and denial of healthcare are forms of slow violence and of abandonment (Davies et al., 2017). Such conditions are endured with no obvious endpoint, as individuals encounter the violence of uncertainty (Grace et al., 2018), living in fear of a return to violence. Those whose claims are rejected are abandoned to the streets and further exploited or detained. The everyday deprivations and humiliations encountered within asylum systems enforce a sense of unbelonging (MasGiralt, 2020), constituted by rejection, and a lack of recognition of respondents’ personhood and potential. The slow violence of immigration and asylum systems thus results in further violence and harms.

Al three forms of violence are “everyday” (Yuval-Davis et al., 2017) in that their impact is constantly enacted and lived daily. Building on Canning (2017), we unpick the ways in which asylum and immigration policy minimize women’s autonomy, operating as a form of control leaving women unsafe. The harms inflicted compound previous experiences of violence. The cumulative effects of structural violence exposed survivors to further interpersonal violence and generated health harms. Rather than facilitating refuge, asylum and immigration systems can remove all hope of “safe and peaceful lives” (Jubilut and de Lima Madureira, 2016) for forced migrant survivors, highlighting the failure of systems to offer a durable solution to the vulnerable. We show elsewhere how these harms undermine attempts to integrate once status is received (Phillimore and Cheung, 2021; Phillimore et al., 2022); they can also be transmitted across generations (Fazel et al., 2015).

## Conclusions

Our work highlights the need for immigration and asylum systems that protect-rather than harm-and that attend to the specific protection needs of forced migrant women, many of whom are SGBV survivors. Suggestions for improvement include ending the lead asylum seeker provisions and providing spouse migrants with details of their rights and entitlements at the visa application stage. In terms of asylum, provision of women-only accommodation, interviewers trained to work in gender- and trauma-sensitive ways, post-interview counseling, cash rather than card payments, and levels of assistance equivalent to general welfare support are all needed. The detention of women and children should cease, and women escaping relationships or whose claims are rejected should be housed and provided with food and clothing. Unfortunately, given that many Western countries are becoming more hostile to forced migrants, they are unlikely to improve provisions.

Further work is needed to examine the structural violence to which women are subject. Our study was not intended to be comparative as the nature of our samples differed across the three countries. Although we identify a few differences in experiences, for example that women in Sweden appear to be better protected and supported when escaping abusive relationships and that women from SSA appear to be more likely to be detained in the United Kingdom, comparative work perhaps involving other countries might help us to identify good and bad practices. Such knowledge could further inform the development of special protections for women who currently have the vulnerabilities that they are seeking to escape, routinely compounded.

## Notes

1. Or equivalent form of leave to remain entitling them to apply for a marriage or family visa.

2. See link Sexual and gender based violence in the refugee crisis: from displacement to arrival (SEREDA) - University of Birmingham.

3. https://www.migrationsverket.se/English/Private-individuals/Moving-to-someone-in-Sweden/If-you-have-come-to-Sweden-because-of-ties-and-are-treated-badly.html.
